# An exploratory study to examine intentions to adopt an evidence-based HIV linkage-to-care intervention among state health department AIDS directors in the United States

**DOI:** 10.1186/1748-5908-7-27

**Published:** 2012-04-02

**Authors:** Wynne E Norton

**Affiliations:** 1 Department of Health Behavior, School of Public Health, University of Alabama at Birmingham, Birmingham, AL, USA

**Keywords:** HIV/AIDS, Policy, Adoption, Linkage-to-care, Intervention

## Abstract

**Background:**

Widespread dissemination and implementation of evidence-based human immunodeficiency virus (HIV) linkage-to-care (LTC) interventions is essential for improving HIV-positive patients' health outcomes and reducing transmission to uninfected others. To date, however, little work has focused on identifying factors associated with intentions to adopt LTC interventions among policy makers, including city, state, and territory health department AIDS directors who play a critical role in deciding whether an intervention is endorsed, distributed, and/or funded throughout their region.

**Methods:**

Between December 2010 and February 2011, we administered an online questionnaire with state, territory, and city health department AIDS directors throughout the United States to identify factors associated with intentions to adopt an LTC intervention. Guided by pertinent theoretical frameworks, including the Diffusion of Innovations and the "push-pull" capacity model, we assessed participants' attitudes towards the intervention, perceived organizational and contextual demand and support for the intervention, likelihood of adoption given endorsement from stakeholder groups (*e.g*., academic researchers, federal agencies, activist organizations), and likelihood of enabling future dissemination efforts by recommending the intervention to other health departments and community-based organizations.

**Results:**

Forty-four participants (67% of the eligible sample) completed the online questionnaire. Approximately one-third (34.9%) reported that they intended to adopt the LTC intervention for use in their city, state, or territory in the future. Consistent with prior, related work, these participants were classified as LTC intervention "adopters" and were compared to "nonadopters" for data analysis. Overall, adopters reported more positive attitudes and greater perceived demand and support for the intervention than did nonadopters. Further, participants varied with their intention to adopt the LTC intervention in the future depending on endorsement from different key stakeholder groups. Most participants indicated that they would support the dissemination of the intervention by recommending it to other health departments and community-based organizations.

**Conclusions:**

Findings from this exploratory study provide initial insight into factors associated with public health policy makers' intentions to adopt an LTC intervention. Implications for future research in this area, as well as potential policy-related strategies for enhancing the adoption of LTC interventions, are discussed.

## Introduction

Timely linkage of newly diagnosed human immunodeficiency virus (HIV)-positive individuals to healthcare is critical for improving patient health outcomes and reducing transmission of HIV to uninfected others [1-4]. Unfortunately, approximately 20% to 40% of newly diagnosed HIV-positive patients in the United States fail to be linked to care in a timely fashion [2,4-6]. Researchers have identified numerous individual-, contextual-, and structural-level factors associated with delayed entry to HIV care [4,7-20]; importantly, such information has served as the foundation for the development of several linkage-to-care (LTC) interventions [21-27].

Despite the potential to improve patient and public health outcomes [1-4,28,29], existing evidence-based HIV LTC interventions have yet to be widely and systematically disseminated and implemented in community-based organizations (CBOs) and health departments throughout the United States. In order to accelerate national-level scale-up, it is important to identify and understand factors associated with intentions to adopt LTC interventions and use this information to develop and test strategies to encourage intervention adoption and effective implementation among key end users, including consumers, organizations, and policy makers.

While limited empirical evidence exists regarding barriers to adoption and effective implementation of LTC interventions (see [30] for a notable exception), related work with evidence-based HIV prevention interventions has identified a range of influential factors, including characteristics of the intervention, organizational culture and climate, program champions, financial resources, and staffing [31-45]. For example, Miller [46] found organizational commitment, resources, and maturity to be distinguishing factors associated with the degree to which an HIV prevention program was adopted in 38 CBOs. Additionally, DiFranceisco and colleagues [31] identified individual- and organizational-level factors associated with attitudes towards using research-based HIV prevention interventions among a sample of 77 AIDS service organizations in the United States.

Comparatively less empirical work, however, has focused on understanding factors associated with intentions to adopt HIV interventions among city, state, and territory policy makers (so-called small p policy makers [47,48]), including state, city, and territory health department AIDS directors. Indeed, health department AIDS directors play a critical role in the dissemination and implementation of interventions in their state, city, or territory, as they are able to actively promote and endorse the adoption of particular interventions within their coverage area or allocate funds for the implementation of particular interventions. Indeed, little is known about factors that influence policy makers' decision-making process regarding the adoption of evidence-based HIV-focused interventions, including LTC interventions.

Given the paucity of research on factors affecting health department AIDS directors' intentions to adopt evidence-based HIV-focused interventions, combined with their "gatekeeper" status and potential role in accelerating dissemination efforts, the objectives of the present study were two-fold: (1) to characterize health department AIDS directors' overall intentions to adopt an evidence-based LTC intervention for use in their state, city, or territory and (2) to compare responses between participants classified as "adopters" versus "nonadopters" on key theory-based constructs.

The current study focused on the adoption of a specific evidence-based LTC intervention, ARTAS (*i.e*., Antiretroviral Treatment Access Study) [19,22,23]. Briefly, ARTAS is a multisession, strengths-based case management [49,50] intervention designed to link newly diagnosed HIV-positive individuals to HIV medical care. In the ARTAS intervention, newly diagnosed HIV-positive individuals meet with a case manager up to five times over a 90-day period. During these sessions, the case manager builds rapport with the client, helps the client identify barriers toward accessing HIV medical care, discusses strategies for overcoming these barriers, and supports the client in seeking HIV medical care. We opted to focus specifically on ARTAS because it is the *only *LTC intervention to date that has demonstrated positive outcomes in both an efficacy and an effectiveness trial [19,22,23], thus providing strong evidence to warrant its widespread use.

## Methods

### Participants

Eligible participants included the 65 health department AIDS directors from all 50 US states, US territories (n = 8; American Samoa, Guam, Marshall Islands, Micronesia, Northern Mariana Islands, Palau, Puerto Rico, and Virgin Islands), the District of Columbia, and six major US cities that receive direct funding from the Centers for Disease Control and Prevention (CDC; *i.e*., Chicago, Houston, Los Angeles, New York, Philadelphia, and San Francisco). The study was approved by the Institutional Review Board at the University of Alabama at Birmingham.

### Procedure

Contact information for each of the 65 AIDS directors was obtained from the online directory of the National Alliance of State and Territorial AIDS Directors (NASTAD; http://www.nastad.org), a nonprofit national association of state and territory health department HIV/AIDS program directors, and supplemented with health department websites, as needed. Individuals were invited to participate in the study via email, which included a link to a 30-minute questionnaire administered through Survey Monkey (http://www.surveymonkey.com, 2011). Data collection occurred from December 2010 to February 2011. The questionnaire included survey items, as well as a brief description of ARTAS (*e.g*., content, purpose, prior research evidence, and recommended staffing) in order to facilitate informed decision making. If directors felt that they were not the most appropriate person to complete the questionnaire, they were asked to forward the study invitation to an appropriate representative within the health department (*e.g*., AIDS prevention coordinator/program manager). Participants received email and/or phone reminders each week for up to three weeks after initial contact and were provided the option of receiving a $30 Visa gift card for participation in the study.

### Measures

In absence of a standardized questionnaire designed specifically for our target population (*e.g*., health department AIDS directors) and evidence-based intervention (*e.g*., LTC intervention), we drew on several theoretical frameworks to inform the selection of key constructs in our questionnaire [47,51,52]. Guided by Rogers' Diffusion of Innovations Theory [52], we included measures to assess participants' attitudes towards the intervention, since attributes of the innovation are associated with innovation adoption [53]. The Diffusion of Innovations Theory also informed our selection of measures to assess participants' likelihood of adoption given endorsement from key stakeholder groups, which served as a proxy for assessing communication channels [52] through which an innovation may be received or promulgated, again influencing end-user adoption. Consistent with Orleans' push-pull capacity model [51,54], we also included constructs to assess health department AIDS directors' perceived demand and support for the LTC intervention, as "pull" factors can hinder or facilitate the adoption of innovations. Validated measures from prior studies [53,55-58] were adapted for the specific context of the present study and used to assess the above-mentioned constructs. Finally, we solicited expert review of the questionnaire from key representatives and researchers from CDC, NASTAD, and academia in an effort to bolster the reliability and validity of our findings given the dearth of validated measures in evidence-based public health policy research.

### Demographic items

Demographic items included age, gender, race/ethnicity, and educational attainment. Participants were asked to identify their current job position (*e.g*., health department director, health department prevention coordinator, other), years in current position, and years worked for the health department. Categorical or continuous response options were provided, as appropriate. These items were selected based on use in a prior study examining the dissemination of physical activity programs among state health departments in the United States [55].

### Intention to adopt ARTAS

Directors' intentions to adopt ARTAS for use in their city, state, or territory was assessed by a single item, "I will adopt ARTAS for use in my city, state, or territory," and measured on a five-point Likert scale (1 = strongly disagree, 5 = strongly agree).

### Attitudes towards ARTAS

Directors' attitudes towards ARTAS were assessed on a 17-item scale. Consistent with the Diffusion of Innovations Theory [52], we assessed participants' attitudes towards attributes of ARTAS, including its relative advantage (*i.e*., degree to which an innovation is perceived as better than the idea it supersedes [52]), complexity (*i.e*., degree to which an innovation is perceived as difficult to understand or use [52]), compatibility (*i.e*., degree to which an innovation is perceived as being consistent with the existing values, past experiences, and needs of potential adopters [52]), observability (*i.e*., degree to which the results of an innovation are visible to others [52]), and trialability (*i.e*., degree to which an innovation may be experimented with on a limited basis [52]). We also assessed participants' attitudes towards intervention effectiveness (*i.e*., performance [59]), cost (*i.e*., money or other resources [59]), and simplicity (*i.e*., inverse of complexity [59]) based on our review of the literature [53,56,58,59]. Sample items include, "Even though ARTAS was shown to be effective in research trials, it wouldn't really work in my city, state, or territory," "ARTAS would have a visible and substantial impact on the health status of newly diagnosed HIV-positive individuals in my city, state, or territory," and "ARTAS is too complex" (reverse scored). Items were assessed on a five-point Likert scale (1 = strongly disagree, 5 = strongly agree). Due to high inter-item correlation, we created a summed scale of attitudes towards ARTAS, with higher scores indicating more positive attitudes towards ARTAS. The scale demonstrated good reliability (α = 0.85).

### Perceived demand and support for ARTAS

Directors' perceptions of demand and support for ARTAS were assessed on a nine-item scale. Consistent with the push-pull capacity model [51], we included items to assess perceived demand for ARTAS among potential end-user organizations (*e.g*., CBOs, local health departments), members of the target population (*e.g*., HIV-positive individuals), and the director's own health department. Sample items include, "There would be a high demand for ARTAS by community-based organizations in my city, state, or territory" and "Linking newly diagnosed HIV-positive individuals to medical care is a high priority in my health department." Items assessed perceived support for ARTAS among political leaders (*e.g*., governor/mayor and legislature/city council) and health department staff (*e.g*., administrators and managers). Sample items include, "The governor of my state or territory/mayor of my city would NOT support the use of ARTAS" and "The state and territory legislature/city council would NOT support the use of ARTAS" (reverse scored). All items were assessed on a five-point Likert scale (1 = strongly disagree, 5 = strongly agree). We created a summed scale of perceived demand and support for ARTAS, with higher scores indicating greater perceived demand and support. The scale demonstrated relatively poor reliability (α = 0.51).

### Likelihood of adoption given endorsement from stakeholders

Fourteen items were used to assess directors' likelihood of adopting ARTAS based on recommendations and endorsement from a variety of key stakeholder groups, as a proxy for assessing communication channels [52]. We selected a range of professional, peer, and nationally based stakeholder organizations involved in HIV care and treatment that would be potential marketing channels for the dissemination of ARTAS in the future. These key stakeholder groups included (1) other states, cities, or territories in the same region; (2) other health department AIDS directors; (3) academic researchers; (4) CBOs; (5) health departments; (6) professional organizations (*e.g*., HIV Medicine Association); (7) councils/groups (*e.g*., National Minority AIDS Council); (8) NASTAD; (9) activist organizations (*e.g*., Community HIV/AIDS Mobilization Project); (10) reputable, online websites (*e.g*., http://www.thebody.com); (11) US government's National AIDS Strategy; (12) Health Resources and Services Administration (HRSA); (13) Veterans Administration (VA); and (14) CDC. Each item asked, "If ARTAS was recommended and endorsed by (name of stakeholder group), I would adopt it for use in my state, city, or territory." Responses were assessed individually on a five-point Likert scale (1 = strongly disagree, 5 = strongly agree).

### Enabling intervention dissemination

Three items were used to assess whether or not directors intended to enable future dissemination efforts by recommending ARTAS to other key stakeholders, including (1) other health department AIDS directors; (2) CBOs within their city, state, or territory; and (3) local health departments within their state or territory (as applicable). Each item stated, "I would recommend ARTAS to (name of key stakeholder group)." Each item was assessed individually by stakeholder group on a five-point Likert scale (1 = strongly disagree, 5 = strongly agree).

### Data analysis

Consistent with the first objective of our exploratory study, descriptive statistics were used to characterize the overall sample in terms of demographics. Descriptive statistics were also used to characterize responses to individual items assessing directors' attitudes towards ARTAS, perceived demand and support for ARTAS, likelihood of adopting ARTAS given endorsement and recommendation from others, and likelihood of enabling intervention dissemination. Inter-item correlations and scale reliability were examined within measures; average scores were computed for scales, where appropriate. Consistent with the second objective of our exploratory study, we compared responses between adopters versus nonadopters on key constructs. Following the approach used in prior research [55], participants were classified as adopters if they responded agree or strongly agree to the single item assessing adoption intentions; those who indicated any other response option (*i.e*., neither agree nor disagree, disagree, and strongly disagree) were classified as nonadopters. One-way independent samples *t*-tests were conducted to examine differences between adopters and nonadopters on demographic variables and average scores (where applicable). All analyses were conducted in SPSS v. 17 (SPSS, Inc., Chicago, IL, USA).

## Results

### Participant characteristics

Table 1 summarizes demographic characteristics of the overall sample. Of 65 eligible participants, 47 agreed to participate (72%), and 42 provided complete responses to all items (64%). Of the five individuals who provided incomplete responses, two initiated the survey but indicated that they were already implementing an LTC intervention, and thus declined to answer any questions because they did not think it was applicable. Two other participants completed the majority of the questionnaire but failed to answer the last few questions. One individual provided his/her name, but did not answer any of the survey items. Thus, analyses for the present study were restricted to 44 individuals who provided responses to the majority of the questionnaire, representing approximately 67% of the total eligible sample.

**Table 1:** Demographic characteristics of participants (N = 44)

Variable	N (%)
Gender	18 (40.9%)
Male	24 (54.5%)
Female	

Race/ethnicity	31 (75.6%)
White	10 (24.3%)
Nonwhite ^a^	

Age (years)	3 (6.8%)
25-35	11 (25.0%)
36-45	18 (40.9%)
46-55	10 (22.7%)
56-65	

Educational degree(s)	6
High school diploma/GED	20
BA/BS	26
MA/MS	7
MD/PhD (or equivalent)	6
Other	

Current position	29 (67.4%)
AIDS Director	9 (20.9%)
AIDS Prevention Program Coordinator	5 (11.6%)
5 (11.6%)	

Years in current position	26 (59.1%)
Less than 5	7 (15.9%)
5-10	7 (15.9%)
11-15	2 (4.7%)
16 or more	

Years in health department	13 (29.5%)
Less than 5	9 (20.5%)
5-10	9 (20.5%)
11-15	(29.5%)
16 or more	

^a^Collapsed due to small cell sizes; includes Native American (n = 1), Pacific Islander (n = 4), Hispanic/Latino (n = 2), and African American (n = 3).

Most participants were white (n = 31, 70.5%), female (n = 24, 54.5%), and between 46 and 55 years old (n = 18, 40.9%). Many participants reported having at least a bachelor's degree (n = 20); several also reported having a Master's degree (n = 26). The majority of participants self-identified as the health department AIDS director (n = 29, 67.4%), while a few participants identified as the department AIDS prevention coordinator/program manager (n = 9, 20.9%) or other (*e.g*., director of policy, director of evaluation, or LTC coordinator; n = 5, 11.7%). Most participants had been in their current position for less than five years (n = 26, 59.1%).

### Intentions to adopt ARTAS

Among the 43 participants who provided a response to the item that assessed intentions to adopt ARTAS, 15 (34.9%) responded agree or strongly agree and were subsequently classified as adopters. Consistent with prior work [55], the remaining 28 participants (65.1%) who responded strongly disagree, disagree, or neither agree nor disagree were classified as nonadopters for between-group analyses.

### Attitudes towards ARTAS

Descriptive statistics and differences between adopters and nonadopters on each individual item assessing attitudes towards ARTAS can be found in Additional file 1: Appendix A. On average, participants classified as adopters reported more positive attitudes towards ARTAS (M = 3.62, standard deviation [SD] = 0.40) than those classified as nonadopters (M = 3.21, SD = 0.32), *t*(41) = 3.65, *p *= .001.

### Perceived demand and support for ARTAS

Descriptive statistics and differences between adopters and nonadopters on each individual item assessing perceived demand and support for ARTAS can be found in Additional file 1: Appendix B. On average, participants classified as adopters reported greater perceived demand and support for ARTAS (M = 3.91, SD = 0.40) than those classified as nonadopters (M = 3.66, SD = 0.29), *t*(41) = 2.38, *p *= .02. These findings should be interpreted with caution, however, given poor scale reliability (α = .51).

### Likelihood of adoption given endorsement from stakeholders

Overall, as displayed in Table 2 a greater percentage of participants reported intentions to adopt ARTAS in the future if ARTAS was recommended and endorsed by CDC (59.1%), the US National HIV/AIDS Strategy (59.1%), HRSA (56.8%), NASTAD (54.5%), or a state, city, or territory health department AIDS director whom the participant respected and trusted (50%). Conversely, a lower percentage of participants reported intentions to adopt ARTAS in the future if ARTAS was recommended and endorsed by reputable online websites (18.2%), VA (25%), or activist organizations (25.6%).

**Table 2:** Descriptive characteristics of intention to adopt ARTAS given endorsement from various stakeholder groups (N = 44)

Item	M	SD	Agree or strongly agree (%)
*If ARTAS was recommended and endorsed by [**stakeholder group**], I would adopt it for use in my state, city, or territory*.			
***Stakeholder groups:***			
... other departments in my geographic region	3.34	0.56	34.1%
... a state, city, or territory AIDS director who I respected and trusted	3.56	0.69	50.0%
... academic researchers	3.45	0.66	40.9%
... community-based organizations that have used it in the past	3.36	0.65	36.4%
... health departments that have used it in the past	3.50	0.62	47.7%
... professional organizations (*e.g*., HIV Medicine Association)	3.25	0.62	30.2%
... HIV/AIDS-related councils or groups (*e.g*., National Minority AIDS Council)	3.29	0.63	34.1%
... National Alliance of State and Territory AIDS Directors (NASTAD)	3.68	0.77	54.5%
... activist organizations (*e.g*., Community HIV/AIDS Mobilization Project)	3.23	0.64	25.6%
... reputable, online websites (*e.g*., www.thebody.com)	3.11	0.57	18.2%
... as part of the National AIDS Strategy	3.75	0.78	59.1%
... Health Resources and Services Administration (HRSA)	3.65	0.77	56.8%
... Veterans Administration (VA)	3.22	0.64	25.0%
... Centers for Disease Control and Prevention (CDC)	3.77	0.80	59.1%

Note: 1 = strongly disagree, 2 = disagree, 3 = neither agree nor disagree, 4 = agree, 5 = strongly agree.

ARTAS = Antiretroviral Treatment Access Study.

### Enabling intervention dissemination

Most participants reported that they would recommend ARTAS to other health departments (56.8%); CBOs in their city, state, or territory (54.5%); and local health departments in their state or territory (61%), respectively.

## Discussion

In the current sample of city, state, and territory health department AIDS directors in the United States, 34.9% reported that they would adopt ARTAS for use in their city, state, or territory. Consistent with prior research [55,60] and several theoretical frameworks [51,52], participants classified as adopters indicated more positive attitudes towards the LTC intervention and greater perceived demand for ARTAS than those classified as non-adopters. Intentions to adopt ARTAS varied by the type of stakeholder group recommending and endorsing the intervention; greater likelihood of adoption was observed when the LTC intervention was hypothetically endorsed by the National HIV/AIDS Strategy and CDC than if endorsed by reputable online websites and VA. Finally, most participants reported that they would recommend ARTAS to other health departments, CBOs, and local health departments (where applicable).

Findings from this exploratory questionnaire study provide an initial description and identification of theory-based constructs associated with intentions to adopt an evidence-based LTC intervention among city, state, and territory health department AIDS directors throughout the United States. Importantly, future research may focus on developing strategies to increase positive attitudes toward the intervention, create demand and support for the intervention, and leverage particular stakeholder groups as effective intervention marketing channels in order to encourage and accelerate the adoption of LTC interventions among city, state, and territory health department AIDS directors.

While the current study provides an initial step towards better understanding policy makers' decision-making processes regarding the adoption of LTC interventions, additional work is needed to develop this area of inquiry further. Indeed, although evidence-based public health policy research has gained momentum in the past decade, especially in the areas of tobacco, cancer, physical activity, and school health [47,61,62], the field would benefit from programmatic efforts to advance both theory and measurement across a variety of public health areas. In the interim, the existing body of work can serve as a starting point for the development of more robust evidence-based public health policy research with specific application towards HIV prevention, treatment, and care interventions.

Several limitations of the current study should be noted. First, responses may be subject to social desirability bias. While procedural safeguards were implemented to reduce such bias (*e.g*., online questionnaire completed at the discretion of the participant's availability and location), it is possible that participants may have provided less-than-accurate responses because the survey was not anonymous. However, having an independent group (*e.g*., academic-affiliated investigator) administer the online survey--rather than an entity charged with allocating funds (*e.g*., CDC, HRSA)--may have reduced the likelihood of biased responses. Second, as is common across most areas of dissemination and implementation research [63], and particularly within evidence-based public health policy research [47,63], the present study lacked established measures for assessing city, state, and territory policy makers'--specifically health department AIDS directors'--intentions to adopt ARTAS in the future. While constructs in the current study were informed by theoretical frameworks; measures used in related-research studies [47,51,52,60,62,64]; and input from expert representatives from CDC, NASTAD, and academia, future work is needed to validate these measures with other HIV-focused interventions and to determine their predictive utility in discriminating between those who actually adopt versus do not adopt interventions in the future.

Identifying ways to promote the adoption of evidence-based LTC interventions among policy makers has the potential to accelerate the transition of such interventions from research to practice settings at the national level, which is essential for realizing the population benefits of our research investments. Although exploratory, the present study contributes to the evidence base in this area and seeks to instigate additional inquiry into this field in a concerted effort to spread evidence-based LTC interventions more quickly and effectively throughout the United States.

## Endnote

^a^For some analyses, N = 43 because of incomplete data or refusal to answer a question.

## Competing interests

The author declares that they have no competing interests.

## Supplementary Material

Additional file 1 **Appendix A**. Descriptives and Differences between Non-adopters (n = 28) and Adopters (n = 15) on Attitudes Towards *ARTAS*. **Appendix B**. Descriptives and Differences between Non-adopters (n = 28) and Adopters (n = 15) on Perceived Organizational and Contextual Demand and Support. Click here for file
