# Supplementary material for: An exploratory study to examine intentions to adopt an evidence-based HIV linkage-to-care intervention among state health department AIDS directors in the United States
Source: Implement Sci. 2012 Apr 2;7:27. doi: 10.1186/1748-5908-7-27 (PMC3348078; doi:10.1186/1748-5908-7-27)
Supplement: Additional file 1 — Appendix A. Descriptives and Differences between Non-adopters (n = 28) and Adopters (n = 15) on Attitudes Towards ARTAS. Appendix B. Descriptives and Differences between Non-adopters (n = 28) and Adopters (n = 15) on Perceived Organizational and Contextual Demand and Support. [file 1748-5908-7-27-S1.DOC]

**Appendix A. Descriptives and Differences between Non-adopters (n = 28) and Adopters (n = 15) on Attitudes Towards *ARTAS***

| Item | Overall  M | Overall  SD | Non-Adopters  M (*SD*) | Adopters  M (*SD*) | Test Statistic |
| --- | --- | --- | --- | --- | --- |
| *ARTAS* would be *more* effective than interventions we are currently using to link newly-diagnosed HIV-positive patients to medical care. | 3.36 | 0.78 | 3.14 (.65) | 3.87 (.74) | t (41) = 3.31, *p* = .002 |
| *ARTAS* is too complex.* | 2.72 | 0.69 | 2.78 (.56) | 2.60 (.91) | t (41) = .83, *p* = .414 |
| *ARTAS* would be successful in linking newly-diagnosed HIV-positive individuals to medical care in my city, state, or territory. | 3.70 | 0.66 | 3.46 (.51) | 4.13 (.74) | t (41) = 3.49, *p* = .001 |
| *ARTAS* is compatible and consistent with the needs of newly-diagnosed HIV-positive individuals in my city, state, or territory. | 3.61 | 0.65 | 3.46 (.58) | 3.93 (.70) | t (41) = 2.35, *p* = .02 |
| *ARTAS* requires too many human resources.* | 2.97 | 0.84 | 3.21 (.78) | 2.53 (.83) | t (41) = 2.65, *p* = .01 |
| *ARTAS* is too expensive.* | 2.95 | 0.68 | 3.14 (.59) | 2.60 (.73) | t (41) = 2.83, *p* = .01 |
| *ARTAS* would be easy to understand and use after receiving training. | 3.75 | 0.57 | 3.64 (.49) | 3.87 (.64) | t (41) = 1.28 *p* = .21 |
| *ARTAS* would have a visible and substantial impact on the health status of newly-diagnosed HIV-positive individuals in my city, state, or territory. | 3.47 | 0.59 | 3.29 (.53) | 3.87 (.52) | t (41) = 3.44, *p* = .001 |
| Newly-diagnosed HIV-positive patients would really benefit from *ARTAS*. | 3.79 | 0.59 | 3.64 (.56) | 4.13 (.52) | t (41) = 2.81, *p* = .007 |
| *ARTAS* could be tested on a trial basis without committing to full implementation. | 3.79 | 0.63 | 3.89 (.50) | 3.60 (.83) | t (41) = -1.45, *p* = .15 |
| If federal funds were NOT available, my health department would pay for *ARTAS* to be used in my city, state, or territory. | 2.25 | 0.99 | 2.07 (.90) | 2.67 (1.05) | t (41) = 1.95, *p* = .06 |
| *ARTAS* could be easily adapted to fit the needs of community-based organizations and/or health departments that would be implementing it. | 3.50 | 0.66 | 3.43 (.50) | 3.60 (.91) | t (41) = .80, *p* = .43 |
| It would be difficult to adapt *ARTAS* to meet the needs of different newly-diagnosed HIV-positive populations.* | 2.59 | 0.58 | 2.75 (.51) | 2.33 (.61) | t (41) = 2.35, *p* = .02 |
| *ARTAS* would be problematic because we do not have enough HIV medical and supportive care resources to care for any additional HIV-positive patients. * | 2.81 | 1.00 | 2.75 (.92) | 2.33 (.61) | t (41) = -.11, *p* = .91 |
| I would *only* be interested in adopting *ARTAS* if funding was provided for it.* | 3.29 | 1.00 | 3.53 (.88) | 2.80 (1.08) | t (41) = 2.41, *p* = .02 |
| Even though *ARTAS* was shown to be effective in research trials, it wouldn’t really work in my city, state, or territory.* | 2.36 | 0.65 | 2.53 (.50) | 2.00 (.75) | t (41) = 2.77, *p* = .008 |
| *ARTAS* would be less effective at linking patients to care in my city, state, or territory than it was in the original research studies.* | 2.56 | 0.69 | 2.75 (.51) | 2.20 (.86) | t (41) = 2.62, *p* = .01 |
| Average score of intervention characteristic items** | 3.35 | .40 | 3.21 (.32) | 3.62 (.40) | t (41) = 3.65, *p* = .001 |

*Note. 1 = Strongly Disagree, 2 = Disagree, 3 = Neither Agree nor Disagree, 4 = Agree, 5 = Strongly Agree*.

*Items were reverse scored prior to conducting t-tests and creation of average score. **Higher score indicates more positive attitudes towards *ARTAS*.

Appendix B. Descriptives and Differences between Non-adopters (n = 28) and Adopters (n = 15) on Perceived Organizational and Contextual Demand and Support

| Item | Overall  *M* | Overall  *SD* | Non-Adopters  M (SD) | Adopters  M (*SD*) | Test Statistic,  *p*-value |
| --- | --- | --- | --- | --- | --- |
| Linking newly-diagnosed HIV-positive individuals to medical care is a high priority for my health department. | 4.65 | 0.88 | 4.86 (.36) | 4.53 (1.06) | t (41) = -1.48, *p* = .15 |
| Administrators and managers at my health department are interested and supportive of evidence-based recommendations to make decisions about adopting HIV interventions. | 4.47 | 0.59 | 4.50 (.58) | 4.47 (.64) | t (41) = -.17, *p* = .86 |
| When it comes to dollars spent specifically for HIV/AIDS, I have the authority to decide whether our health department will adopt a particular HIV intervention. | 3.70 | 1.02 | 3.54 (1.04) | 4.07 (.96) | t (41) = 1.64, *p* = .11 |
| There would be a high demand for *ARTAS* by community-based organizations in my city, state, or territory. | 3.22 | 0.74 | 3.07 (.66) | 3.53 (.83) | t (41) = 1.99, *p* = .05 |
| There would be a high demand for *ARTAS* by local (city or county) health departments in my city, state, or territory. | 3.04 | 0.71 | 2.86 (.52) | 3.40 (.91) | t (41) = 2.49, *p* = .02 |
| The governor of my state or territory / mayor of my city would NOT support the use of *ARTAS*.* | 2.84 | 1.07 | 2.75 (.96) | 3.00 (1.30) | t (41) = 1.13, *p*= .27 |
| The state or territory legislature / city council would NOT support the use of *ARTAS*.* | 2.93 | 0.99 | 2.85 (.84) | 3.06 (1.27) | t (41) = 1.34, *p* = .51 |
| Providing funds to implement *ARTAS* would be a good use of federal dollars that are allocated specifically for HIV/AIDS activities. | 3.88 | 0.81 | 3.82 (.77) | 4.00 (.93) | t (41) = .674, *p* = .50 |
| Newly-diagnosed HIV-positive individuals would be interested in receiving *ARTAS*. | 3.59 | 0.58 | 3.50 (.58) | 3.73 (.59) | t (41) = 1.25, *p* = .22 |
| Average score of support and climate** | 3.75 | .35 | 3.66 (.29) | 3.91 (.40) | t (41) = 2.38, *p* = .02 |

*Note. 1 = Strongly Disagree, 2 = Disagree, 3 = Neither Agree nor Disagree, 4 = Agree, 5 = Strongly Agree*. *Items reversed scored prior to conducting t-test and average measure. **Higher score indicates greater perceived demand and support for *ARTAS*.
